# Genotype–phenotype interaction in Alzheimer's disease immune activation

**DOI:** 10.1002/alz.70978

**Published:** 2026-01-09

**Authors:** Stephanie Knudtzon, Bjørn‐Eivind Kirsebom, Lene Pålhaugen, Francesco Bettella, Berglind Gísladóttir, Shahram Bahrami, Lavinia Athanasiu, Arvid Rongve, Arne Nakling, Ina S. Almdahl, Lisa Kalheim, Jonas Alexander Jarholm, Gøril Rolfseng Grøntvedt, Ragnhild Eide Skogseth, Dag Aarsland, Knut Waterloo, Ole A. Andreassen, Tormod Fladby, Kaja Nordengen

**Affiliations:** ^1^ Department of Neurology University Hospital of North Norway Tromsø Norway; ^2^ Department of Psychology Faculty of Health Sciences UiT The Arctic University of Norway Tromsø Norway; ^3^ Department of Neurology Akershus University Hospital, Lorenskog Lørenskog Norway; ^4^ Institute of Clinical Medicine University of Oslo Oslo Norway; ^5^ Section for Precision Psychiatry Department of Psychiatry Oslo University Hospital Oslo Norway; ^6^ Department of Medical Genetics Oslo University Hospital Oslo Norway; ^7^ Clinical Molecular Biology (EpiGen) Medical Division Akershus University Hospital and University of Oslo Oslo Norway; ^8^ Department of Research and Innovation Haugesund Hospital Haugesund Norway; ^9^ Institute of Clinical Medicine (K1) University of Bergen Bergen Norway; ^10^ Department of Neurology and Clinical Neurophysiology University Hospital of Trondheim Trondheim Norway; ^11^ Department of Neuromedicine and Movement Science Faculty of Medicine and Health Sciences Norwegian University of Science and Technology Trondheim Norway; ^12^ Department of Geriatric Medicine and the Neuro‐SysMed Centre Haraldsplass Deaconess Hospital Bergen Norway; ^13^ Department of Old Age Psychiatry Institute of Psychiatry, Psychology and Neuroscience King's College London London UK; ^14^ Center for Age‐Related Diseases Stavanger University Hospital Stavanger Norway

**Keywords:** Alzheimer's disease, clusterin, fractalkine, phenotype, polygenic risk scores, sTREM2, YKL‐40

## Abstract

**INTRODUCTION:**

Alzheimer's disease (AD) is a neurodegenerative disorder influenced by genetic factors, particularly related to immune activation. This study examines genotype‐phenotype interactions affecting immune responses in AD as a reaction to neurodegeneration.

**METHODS:**

We computed AD polygenic risk scores (PRSs) informed by shared AD‐autoimmunity genetics (AD PRS_INFL_), AD‐independent immune activation score from 10 autoimmune diseases (sum PRS_IMMUNE_), and standard AD PRSs for 294 individuals. Cerebrospinal fluid (CSF) immune markers (sTREM2, clusterin, fractalkine, and chitinase 3 like protein (YKL‐40)) were regressed on PRSs, and their interaction with neurodegeneration markers (total tau [t‐tau] or neurofilament light chain [NfL]).

**RESULTS:**

High AD PRS_INFL_ scores correlated with lower sTREM2 (*β *= −0.18, *p* < 0.01), clusterin (*β *= −0.12, *p* < 0.05), and fractalkine (*β *= −0.13, *p* < 0.05) levels in cases with elevated t‐tau. High sum PRS_IMMUNE_ score correlated with lower clusterin in cases with elevated NfL (*β *= −0.12, *p* < 0.05).

**DISCUSSION:**

Genetic predisposition for immune activation might cause unfavorable immune response in early AD.

**Highlights:**

- High genetic risk relates to cerebrospinal fluid (CSF) immune markers amid evident neurodegeneration
- Immune polygenic risk scores (PRSs) capture genotype‐phenotype associations in Alzheimer's disease (AD) immune activation
- AD PRSs and immune phenotype had an opposite relationship of the phenotype PRSs
- Importance of specific genetic predispositions in early AD immune phenotypes

## 1 INTRODUCTION

Alzheimer's disease (AD) is the leading cause of dementia, being responsible for 60%–80% of the cases.^1^ AD is highly heritable, with ∼70% of its variance estimated to be due to genetics,^2^ but seems to have a complex etiology.^3^, ^4^, ^5^ AD polygenic risk scores (PRSs) have been calculated and refined based on genome‐wide association studies (GWAS).^6^, ^7^, ^8^ AD PRSs predict cognitive decline,^9^ but account for only 24%–33% of the phenotypic variance.^10^, ^11^ Their limited explanatory power may be due to the influence of several unidentified AD genetic risk variants.^12^ In genotype‐phenotype association studies, genetic predispositions may require specific conditions to manifest as observable traits. For example, young individuals with high versus low PRSs for hypertension show little deviation in blood pressure, but differences appear among individuals of increasing age.^13^ PRSs have been shown to improve disease prediction for diabetes type 2, atrial fibrillation, breast cancer, and prostate cancer, with higher PRSs linked to earlier onsets of these diseases.^14^ This underscores the potential of using genetic information to refine our understanding of when a genetic predisposition will manifest as a clinical phenotype.

It is well established that the innate immune system plays an important role in AD susceptibility.^3^, ^15^, ^16^ Focusing on innate immune activation in AD rather than AD risk or age of onset, may help identifying subgroups that are particularly susceptible to altered AD risk‐mediating patterns of immune activation and inflammation. For this purpose, PRSs encompassing risk for autoimmune disease may be used to reveal genetic factors contributing to AD phenotypes. We therefore constructed an AD‐independent PRSs by summing the cumulative risk of 10 autoimmune diseases (sum PRS_IMMUNE_). Additionally, we employed a phenotype informed inflammatory PRSs based on the genetic risk shared by AD and 11 autoimmune diseases (AD PRS_INFL_) previously found to predict medial temporal lobe atrophy and better correlate with cognitive decline and cerebrospinal fluid (CSF) amyloid beta (Aβ) than a pure AD PRS,^7^ but as yet, untested in relation to the corresponding overarching phenotypic outcome, in this case innate immune activation. Earlier studies have shown that many CSF innate immune activation markers in AD are associated with tau‐pathology,^17^, ^18^ highlighting the latter as a promising candidate for exploring interactions between immune‐related genetic risk and core pathologies in the AD continuum.

The aim of the current study was to explore how genetic predispositions (AD PRS, AD PRS_INFL_, and sum PRS_IMMUNE_) might manifest as observable traits in the form of innate immune activation (three anti‐inflammatory markers: soluble triggering receptor expressed on myeloid cell 2 (sTREM2), clusterin and fractalkine and one inflammatory marker: chitinase‐3‐like protein 1 (YKL‐40)) in the presence of neurodegeneration measured as CSF total tau (t‐tau) and neurofilament light chain (NfL), two neurodegeneration markers representing different neuropathological processes.^19^ Building on our earlier study, which found elevated concentrations of sTREM2, clusterin, fractalkine and YKL‐40 in the presence of tau pathology,^17^ we specifically selected these four CSF innate immune activation markers for the current study.

## 2 METHODS

### 2.1 Study design

The Dementia Disease Initiation (DDI) study is a longitudinal multi‐site Norwegian study initiated in 2013.^20^ Participants are recruited through memory clinics, newspaper advertisements, news bulletins, and media, and through spouses of individuals with cognitive disorders. Orthopedic patients with normal cognition undergoing lumbar puncture before surgery are also included. Inclusion criteria are age between 40 and 80 years, and no history of brain trauma or brain disorders, severe psychiatric or somatic disease, or other developmental disorders that might influence cognitive functions or intellectual disability. All participants undergo a standardized research protocol that includes an assessment of patient history, blood and CSF collection, clinical and neurological examination, and neuropsychological testing.

**RESEARCH IN CONTEXT**

1. **Systematic review**: We conducted a focused literature search utilizing PubMed, Google Scholar, and reference lists to gather existing knowledge on polygenic risk scores (PRSs) in Alzheimer's disease (AD). Our search centered on genotype‐phenotype interactions, particularly examining how PRS reveals phenotypes under certain conditions. To extend our context, we also reviewed PRS applications in other diseases, identifying patterns where genetic predispositions manifest only under specific conditions, such as age‐related hypertension. This informed our study on immune‐related genetic factors in AD progression.
2. **Interpretation**: Our findings indicate that specialized PRSs capture relevant genotype–phenotype associations related to immune activation in AD, different from standard AD PRSs.
3. **Future directions**: Future research should consider specific genetic predispositions when evaluating immune activation phenotypes in the preclinical and prodromal stages of AD.

This study´s sample comprised 294 individuals with a mean age of 64.0 years (SD 9.3, 40–80 years), including 160 females (54.4%). Among the participants, 57 (19.4%) were recruited as controls, 98 (33.3%) had subjective cognitive decline (SCD) and 139 (47.3%) had mild cognitive impairment (MCI), see table 1. SCD is defined by the SCD‐I framework^21^ as experiencing a decline in a cognitive domain, but performing normally on objective neuropsychological tests. Participants with MCI, were identified following the NIA‐AA criteria^22^ and scored lower than expected in one or more cognitive domains but were independent in daily functional ability. A total of 130 (44.2%) participants had at least one apolipoprotein E (*APOE) ‐ε4* allele. Only 3.1% (*n* = 9) had autoimmune diseases, while 29.6% (*n* = 87) were regularly treated with non‐steroidal anti‐inflammatory drugs (NSAIDs). 29.9% (*n* = 88) participants were Aβ positive cases, while 1.7% (*n* = 5) had no information concerning their Aβ status. All 294 participants (100%) had data on t‐tau, and of those, 238 (80.9%) had available NfL. Information on CSF sTREM2, clusterin, fractalkine and YKL‐40 was available for 243 (82.7%), 289 (98.3%), 285 (96.9%) and 288 (98.0%) individuals, respectively. We lacked data for 51 (17.3%) individuals on sTREM2, for 5 (1.7%) individuals on clusterin, for 9 (3.1%) individuals on fractalkine and for 6 (2.0%) individuals on YKL‐40.

**TABLE 1:** Demographic of age, sex, *APOE‐ε4* carrier status, autoimmune disease, NSAIDs, Aβ positive cases, neurodegeneration markers, cognitive status, and CSF innate immune activation markers

Total (*n* = 294)	
Age Mean (SD)	64.0 (9.3)
Female sex *n* (%)	160 (54.4)
*APOE‐ε4 n* (%)	130 (44.2)
Autoimmune disease *n* (%)	9 (3.1)
NSAIDs *n* (%)	87 (29.6)
*Aβ* positive cases *n* (%) [*n*]	88 (29.9) [289]
RaC *n* (%)	57 (19.4)
SCD *n* (%)	98 (33.3)
MCI *n* (%)	139 (47.3)
CN *n* (%) A*β* negative / *n* (%) A*β* positive	116 (40.1) / 36 (12.5)
MCI *n* (%) A*β* negative / *n* (%) A*β* positive	85 (29.4) / 52 (17.9)
t‐tau mean (SD)	372.5 (215.6)
NfL mean (SD) [*n*]	3320.8 (1963.6) [238]
sTREM2 mean (SD) [*n*]	4.25 (1.54) [243]
Clusterin mean (SD) [*n*]	2107 (753) [289]
Fractalkine mean (SD) [*n*]	2006 (589) [285]
YKL‐40 mean (SD) [*n*]	173 (70.3) [288]

Abbreviations: %, percentage; APOE‐ε4, apolipoprotein E type 4 allele;Aβ, amyloid beta; CN, cognitive normal (including RaC and SCD); MCI, mild cognitive impairment; *n*, number of cases; NfL, neurofilament light chain; NSAIDs, non‐steroidal anti‐inflammatory drugs; RaC, recruited as controls; SCD, subjective cognitive decline; sTREM2, soluble triggering receptor expressed on myeloid cell 2; t‐tau, total tau; YKL‐40, chitinase‐3‐like protein 1.

### 2.2 Lumbar puncture and CSF markers

Polypropylene tubes (Thermo Fisher Scientific, MA, USA) were used to collect CSF. Collections were done before noon and centrifuged within 4 h at 2000 × *g* for 10 min at room temperature. The samples were then transferred to new tubes and frozen directly at −80°C on site, before being shipped frozen on dry ice to a central lab facility at Akershus University Hospital, where they were was kept at −80°C until analysis. CSF NfL, Aβ_1‐40_, Aβ_1‐42_, sTREM2, clusterin and fractalkine were measured by the QuickPlex SQ 120 system from Meso Scale Discovery (MSD, MD, USA), as previously described in detail.^17^, ^23^ Due to differences in the 9‐Plex and 4‐plex setups for fractalkine, we evaluated potential adjustments due to between‐setup differences in our statistical models by computing a random intercept (linear mixed models) for setup differences. Since the results and model fits were similar, we proceeded without adjustments.^17^ sTREM2 was analyzed using a sandwich enzyme‐linked immunosorbent assay (ELISA) method described by others.^24^ CSF t‐tau was determined using Innotest hTau Ag kit (Fujirebio, Ghent, Belgium). The ratio of CSF Aβ_1‐42_ to Aβ_1‐40_ (Aβ_42/40_ ratio) was used to determine the presence or absence of Aβ plaque pathology. The cut‐off (≤.077) for Aβ_42/40_ ratio was determined using ROC analysis of visual [18F]‐Flutemetamol PET scans readings.^25^ APOE genotyping was performed on ethylenediaminetetraacetic acid (EDTA) blood samples, as previously described.^20^

### 2.3 PRSs

All 294 participants were genotyped with Human Omni Express‐24 v.1.1 (Illumina Inc., San Diego, CA, USA) at deCODE Genetics (Reykjavik, Iceland). High linkage disequilibrium (LD) regions, in the major histocompatibility complex (MHC, human genome build hg19 location chr6:25119106‐33854733) and 8p23.1 (human genome build hg19 location chr8:7200000–12500000), in addition to the extended APOE region (chr19:44909039–45912650) were excluded from all analyses. Standard *p*‐value‐based PRS were computed using PRSice following in essence the method originally described by the International Schizophrenia Consortium.^26^ The PRS of a given individual is the sum across all included single nucleotide polymorphisms (SNPs) of the products of the SNP's estimated effect and the number of copies of the SNP in the individual's DNA. PRSice automates PRS computation ranking SNPs based on their association statistics. Standard *p*‐value‐based PRSs were computed using a recent AD GWAS^27^ and GWASs for autoimmune diseases: ulcerative colitis,^28^ Crohn's disease,^28^ rheumatoid arthritis,^29^ celiac disease,^30^ psoriasis,^31^ multiple sclerosis,^32^ primary sclerosing cholangitis,^33^ systemic lupus erythematosus,^34^ vitiligo^35^ and ankylosing spondylitis.^36^ The sum PRS_IMMUNE_ was calculated as the sum of these individual autoimmune PRSs and is hence independent of the AD GWAS. The AD PRS_INFL_ was constructed using effect estimates from the AD GWAS but selecting SNPs based on their association with AD *conditional* on their association with inflammatory traits, using the conditional false discovery rate (condFDR) approach.^37^ To be considered significantly associated with AD, a variant's condFDR statistic must fall under the threshold (condFDR < 0.01) for at least one of the conditional inflammatory traits. Only lead variants (i.e., variants with lowest condFDR statistic) from the genomic loci singled out by the false discovery rate (FDR) fitting procedures were included in the final scores, computed using PRSice software as described previously.^7^ Both the sum PRS_IMMUNE_ and the AD PRS_INFL_ are derived from the same set of autoimmune diseases, except for diabetes mellitus type 1,^38^ which is excluded from the sum PRS_IMMUNE_ due to lack of GWAS weighting information. Although diabetes mellitus type 1 is not included in the sum PRS_IMMUNE_, it is not expected to have a significant impact on the results as the primary distinction between the sum PRS_IMMUNE_ and AD PRS_INFL_ lies between AD‐dependent and AD‐independent factors. For both the sum PRS_IMMUNE_ and the AD PRS_INFL_ the *p*‐value thresholds were set to 0.01, as previously described.^7^

### 2.4 Statistical analyses

Statistical analyses were conducted in R v4.1.2.^39^ Multiple linear regression models were fitted to estimate the effect of the interaction between PRSs and CSF neurodegeneration markers on CSF innate immune activation markers (CSF innate immune activation markers ∼ PRS * neurodegeneration marker). Additionally, we evaluated the effect of age, sex, NSAIDs, and autoimmune diseases as covariates in our models. To ensure model parsimony, non‐significant covariates were systematically removed through a stepwise process. Based on our previous studies,^23^, ^40^ adjusting for age could inadvertently remove variance directly attributable to the pathology under investigation, potentially obscuring the true relationship between genetics and CSF innate immune activation marker levels. Despite significant, age was therefore excluded from the final models. Nevertheless, results from analyses including age as a covariate are presented as supplementary sensitivity analyses. To assess whether the assumptions of linear regression, such as the normality of residuals and the absence of heteroscedasticity, were met, we examined Q–Q plots and plots of residuals versus predicted values. Due to slight departures from normality in the residual diagnostics, all CSF innate immune activation markers were log‐transformed and standardized prior to the final analyses. To account for the assessment of four CSF innate immune activation markers for each PRS, we applied false discovery rate correction to adjust for multiple testing across the four models. The significance threshold was set at *p* < 0.05 for all analyses.

## 3 RESULTS

### 3.1 AD PRS, CSF innate immune activation, and neurodegeneration markers

A positive association was observed between AD PRS and sTREM2 concentrations with higher CSF NfL concentrations (*β *= 0.15, *p* < 0.05). This association remained statistically significant after adjusting for multiple testing (p_adj _< 0.05). Similarly, a significant result was found between AD PRS, YKL‐40, and NfL (*β *= 0.15, *p* < 0.01, p_adj _< 0.05). No significant main effects or interaction effects were found between AD PRS and neurodegeneration markers (t‐tau or NfL) for neither clusterin nor fractalkine in this study. See supplementary table S1.

### 3.2 AD PRS_INFL_, CSF innate immune activation, and neurodegeneration markers

**FIGURE 1:**
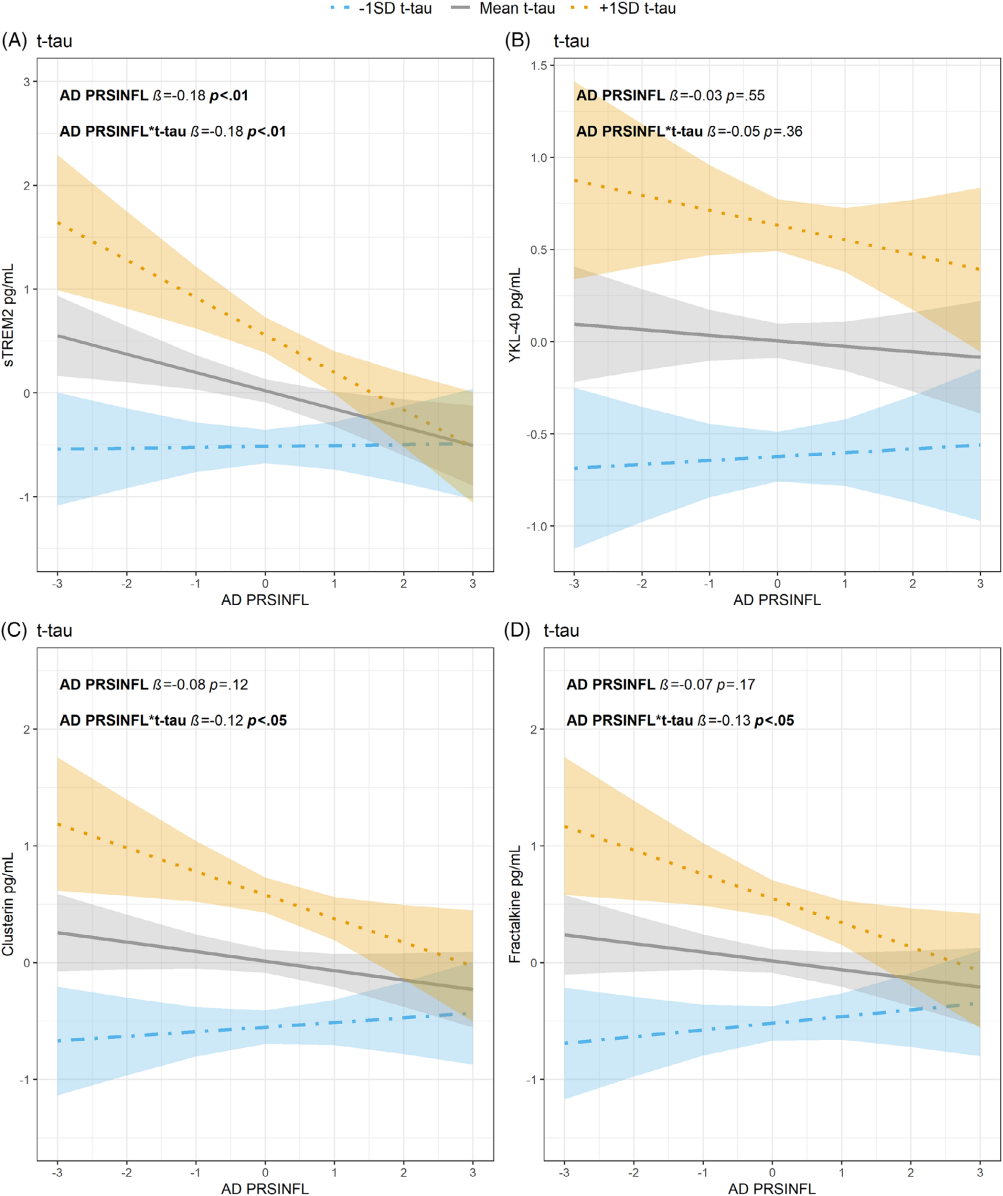
Relationship between t‐tau, AD PRS_INFL_ z‐score (*x*‐axes), and CSF activation marker z‐score (*y*‐axes) (A) sTREM2, (B) YKL‐40, (C) clusterin, and (D) fractalkine. T‐tau values are given as 1 SD level below mean (blue dot‐dashed line, with 95% confidence interval), mean levels (gray line, with 95% confidence interval) or 1 SD level above mean (orange dotted line with 95% confidence interval). CSF, cerebrospinal fluid; PRS, polygenic risk score; SD, standard deviation; sTREM2, soluble triggering receptor expressed on myeloid cell 2; t‐tau, total tau; YKL‐40, chitinase‐3‐like protein 1

With higher CSF t‐tau concentrations a negative association was observed between AD PRS_INFL_ and sTREM2 (*β *= −0.18, *p* < 0.01), and the association remained significant after adjusting for multiple testing (p_adj _< 0.01). A lower AD PRS_INFL_ was associated with higher concentrations of both clusterin and fractalkine as CSF t‐tau concentrations increased (*β *= −0.12, *p* < 0.05 and *β *= −0.13, *p* < 0.05, respectively). However, these associations were not significant after correction for multiple testing (p_adj _= 0.08 and p_adj _= 0.06, respectively). No significant interaction was observed between AD PRS_INFL_, YKL‐40 and t‐tau. See Figure 1 and table S1.

**FIGURE 2:**
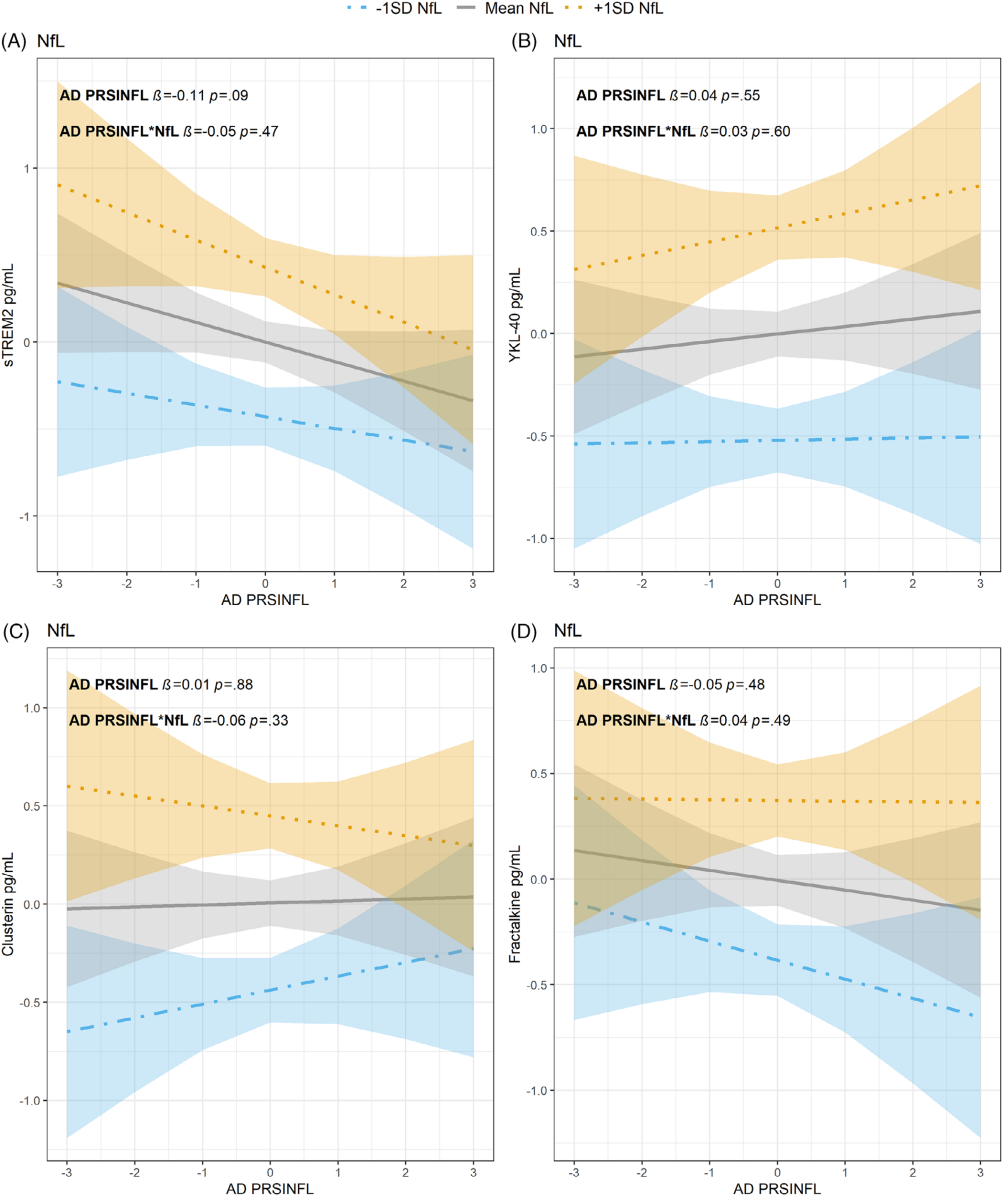
Relationship between NfL, AD PRS_INFL_ *z*‐score (*x*‐axes), and CSF activation marker *z*‐score (*y*‐axes) (A) sTREM2, (B) YKL‐40, (C) clusterin and (D) fractalkine. NfL values are given as 1 SD level below mean (blue dot‐dashed line, with 95% confidence interval), mean levels (gray line, with 95% confidence interval) or 1 SD level above mean (orange dotted line with 95% confidence interval). AD, Alzheimer's disease; CSF, cerebrospinal fluid; NfL, neurofilament light chain; PRS, polygenic risk score; SD, standard deviation; sTREM2, soluble triggering receptor expressed on myeloid cell 2; YKL‐40, chitinase‐3‐like protein 1

There were no significant associations between CSF innate immune activation markers (dependent variables) and the interaction term between the two independent variables AD PRS_INFL_ and NfL concentrations. See Figure 2 and table S1.

### 3.3 Sum PRS_IMMUNE_, CSF innate immune activation, and neurodegeneration markers

**FIGURE 3:**
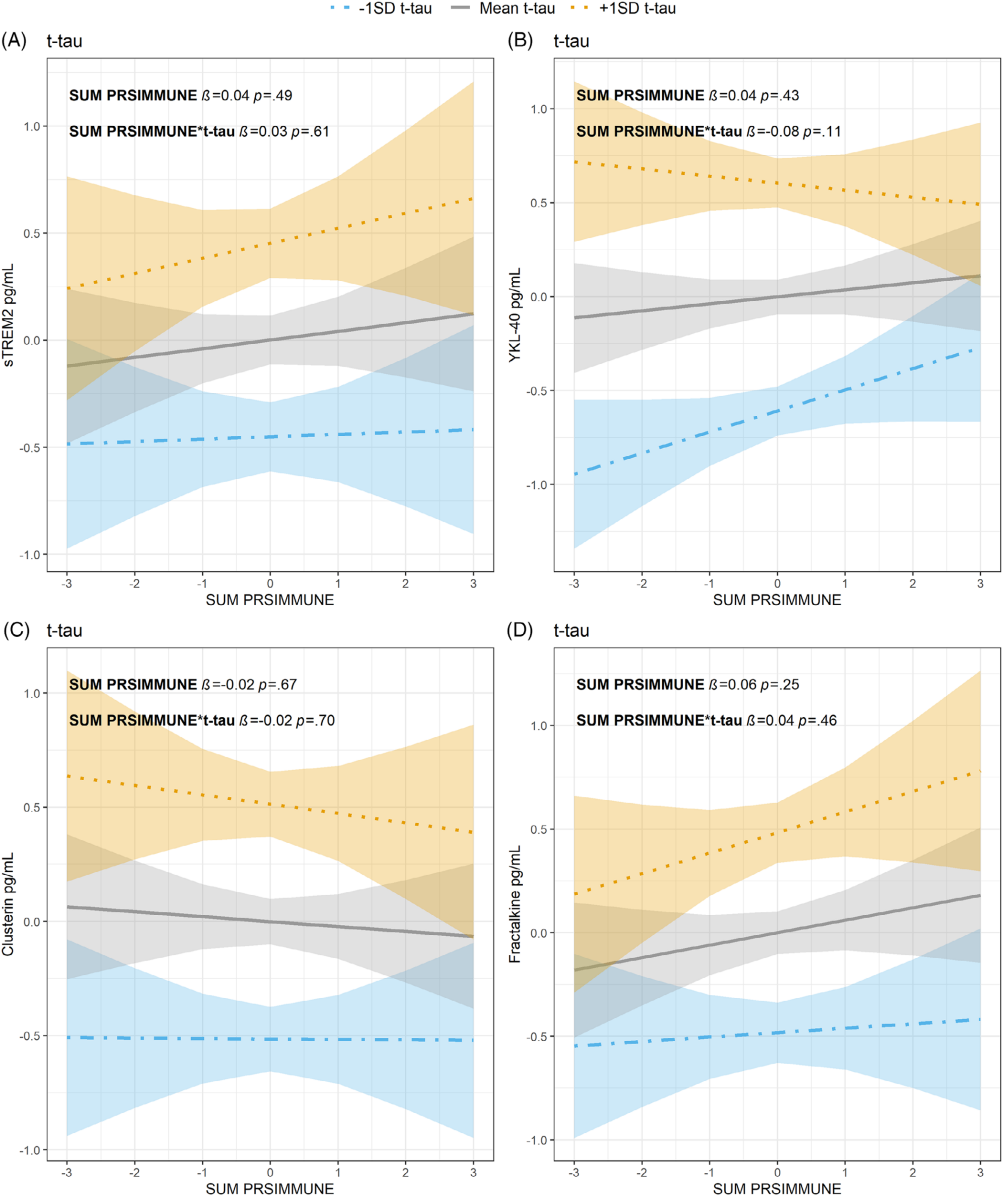
Relationship between t‐tau, sum PRS_IMMUNE_ *z*‐score (*x*‐axes), and CSF activation marker *z*‐score (*y*‐axes) (A) sTREM2, (B) YKL‐40, (C) clusterin, and (D) fractalkine. T‐tau values are given as 1 SD level below mean (blue dot‐dashed line, with 95% confidence interval), mean levels (gray line, with 95% confidence interval) or 1 SD level above mean (orange dotted line with 95% confidence interval). CSF, cerebrospinal fluid; PRS, polygenic risk score; SD, standard deviation; sTREM2, soluble triggering receptor expressed on myeloid cell 2; t‐tau, total tau; YKL‐40, chitinase‐3‐like protein 1

No significant associations between sum PRS_IMMUNE_, CSF innate immune activation markers and t‐tau concentrations were found. See Figure 3, Table S1. Even though the sTREM2 and fractalkine associations between sum PRS_IMMUNE_ and t‐tau were not significant, it is worth noticing the opposite trends observed in sTREM2 and fractalkine concentrations between AD PRS_INFL_ and sum PRS_IMMUNE_. See Figures 1A,D versus 3A,D.

**FIGURE 4:**
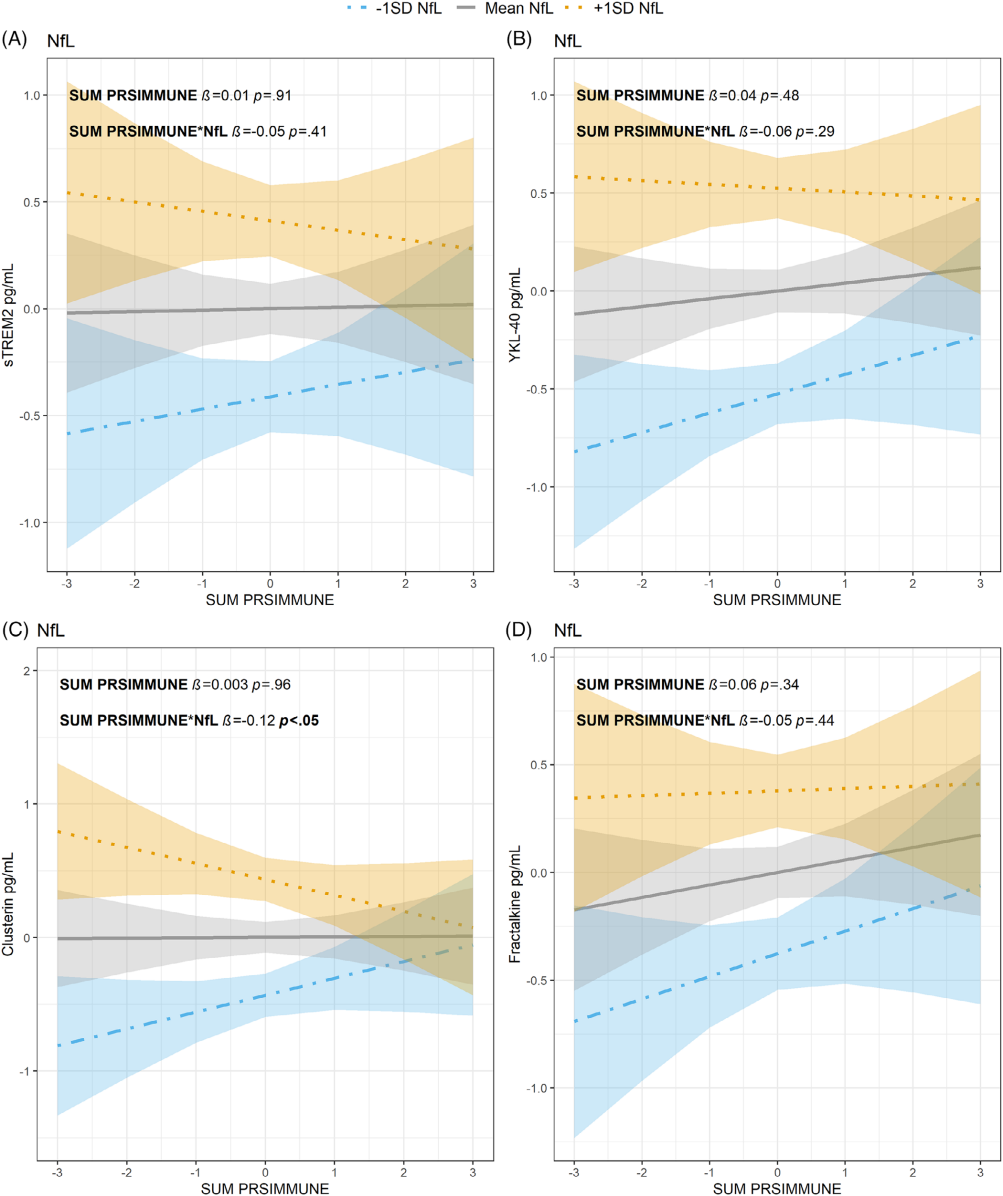
Relationship between NfL, sum PRS_IMMUNE_ *z*‐score (*x*‐axes), and CSF activation marker *z*‐score (*y*‐axes) (A) sTREM2, (B) YKL‐40, (C) clusterin and (D) fractalkine. NfL values are given as 1 SD level below mean (blue dot‐dashed line, with 95% confidence interval), mean levels (gray line, with 95% confidence interval) or 1 SD level above mean (orange dotted line with 95% confidence interval). CSF, cerebrospinal fluid; NfL, neurofilament light chain; PRS, polygenic risk score; SD, standard deviation; sTREM2, soluble triggering receptor expressed on myeloid cell 2; YKL‐40, chitinase‐3‐like protein 1

A higher sum PRS_IMMUNE_ was associated with lower concentrations of clusterin when CSF NfL concentrations were increased (*β *= −0.12, *p* < 0.05). However, the association did not remain significant after correction for multiple testing (p_adj _= 0.08). No significant associations were found between sum PRS_IMMUNE_ and the remaining CSF innate immune activation markers (sTREM2, fractalkine, YKL‐40) and NfL concentrations. See Figure 4 and table S1.

### 3.4 Sum sensitivity analyses

When age was included as a covariate in the analysis, we still did not find any significant main effects or interaction effects between AD PRS, CSF neurodegeneration markers (t‐tau or NfL) and CSF innate immune activation markers. See table S2.

For the AD PRS_INFL_, a negative association with sTREM2 (*β *= −0.15, *p* < 0.05) at higher t‐tau concentrations was observed, and this association remained significant after adjusting for multiple testing (p_adj _< 0.05), although the strength of the negative association was reduced compared to the analyses without age as a covariate. Furthermore, there was a significant negative association between the AD PRS_INFL_ and fractalkine (*β *= −0.12, *p* < 0.05) with high t‐tau concentrations, but it did not remain significant after adjusting for multiple testing (p_adj _= 0.07), same as the analyses without age as a covariate and the strength of the association was almost identical (with age: *β *= −0.12 vs. without age: *β *= −0.13). No other significant associations were found for the AD PRS_INFL_ score. See table S2.

Finally, for the sum PRS_IMMUNE_, a significant association was observed for clusterin and NfL (*β *= −0.11, *p* < 0.05) but did not remain significant after adjusting for multiple testing (p_adj _= 0.06). Otherwise, no other significant association was found between sum PRS_IMMUNE_, CSF innate immune activation markers and CSF t‐tau concentrations or CSF NfL concentrations similar to the analyses without age as a covariate. See table S2.

## 4 DISCUSSION

The present study suggests that genetic predisposition related to innate immune activation manifest as reduced anti‐inflammatory biomarkers in CSF during early neurodegeneration (indicated by relatively increased CSF t‐tau and NfL concentrations). We found that a higher AD PRS_INFL_ was associated with lower CSF sTREM2, clusterin, and fractalkine when CSF t‐tau concentrations were higher, suggesting a lacking anti‐inflammatory immune activation as response to neurodegeneration among those with high AD PRS_INFL_. Low sum PRS_IMMUNE_ was associated with higher CSF clusterin when CSF NfL concentrations were higher, again suggesting a lack of anti‐inflammatory immune activation as response to neurodegeneration among those with high sum PRS_IMMUNE_. However, after adjusting for multiple testing, only the association with sTREM2 and the interaction between t‐tau and AD PRS_INFL_ remained significant. In contrast to both the AD‐dependent and the AD‐independent PRS immune activation scores, we found no associations between the conventional AD PRS and any of the CSF innate immune activation markers.

We previously reported a significant genetic overlap between the AD PRS_INFL_ and the standard AD PRS,^7^ consistent with findings from Tesi et al. (2020),^41^ which showed that immune response contributed to 45.5% of the total AD PRS when APOE variants were excluded from the score. The genotype‐phenotype associations observed with the two new immune activation PRSs (AD PRS_INFL_ and sum PRS_IMMUNE_), which also include polymorphisms not present in the standard AD PRS, suggest that these PRSs capture effects on immune activation in AD that are not captured by the standard AD PRS. These specialized PRSs, hence, appear to provide additional insights into AD‐related immune activation compared to the standard AD PRS, although the latter also entails some aspects of immune activation and genes relevant to this process.

Finding the best PRS for early identification of individuals at risk of developing AD may enable the implementation of preventive measures and/or medication. This is plausible since AD pathology develops 10–15 years before the actual clinical symptoms^42^, ^43^ and predementia stages are detectable through markers in CSF and plasma,^44^, ^45^, ^46^, ^47^ but most importantly since premorbid genetic risk for disease subgroups may be mapped at early preclinical stages prior to expression of AD subgroup phenotypes. We have shown that different PRSs are associated with inadequate response to neurodegeneration marked by lower concentrations of anti‐inflammatory cytokines. This suggests that these new PRSs could serve as potential biomarkers for identifying key subgroups during the early preclinical stages. Furthermore, we have observed that the innate immune activity phenotype generally does not manifest before neurodegeneration is present, with the noted exception of sTREM2.

The TREM2 receptor is expressed and upregulated in microglia surrounding Aβ plaque in AD.^48^ Increased expression of this receptor in mice microglia has been found to be neuroprotective by downregulating genes associated with innate immune activation and upregulating phagocytosis‐related genes.^49^, ^50^ Mutations in TREM2 leads to increased risk of AD.^50^, ^51^, ^52^ When TREM2 is cleaved, sTREM2 is released and measurable in the CSF.^53^ CSF sTREM2 is found to be increased in early stages of AD^54^, ^55^ with increasing tau concentrations,^54^ correlating with microglial activation.^48^ In our study, sTREM2 and AD PRS_INFL_ had a significant negative association. A higher AD PRS_INFL_ was associated with lower sTREM2 concentrations, while higher t‐tau concentrations were associated with higher sTREM2 concentrations. When the interaction term between AD PRS_INFL_ and t‐tau was included in the regression analysis, a higher AD PRS_INFL_ significantly pulled the results in a negative direction. It is tempting to speculate that people with a high AD PRS_INFL_ have lower beneficial response of sTREM2 through life cumulatively exposing them to a higher risk of developing AD due to low Aβ clearance. The lack of a significant association between sTREM2 and AD PRS_INFL_ when adjusting for NfL could be explained by NfL being a more unspecific marker of AD neurodegeneration compared to t‐tau.^56^, ^57^

In our primary models we did not adjust for age. In this mixed 40–80‐year sample comprising both cognitively intact and impaired individuals, age may inadvertently index the probability/stage of neurodegenerative disease and including it as a covariate may reallocate pathology‐related variance from t‐tau/NfL to age, thereby attenuating the estimated coupling between neurodegeneration and the immune response (sTREM2). In a previous study, we showed that age‐adjusting CSF t‐tau and NfL using normative models reallocated disease‐related variance to the age term, producing over‐adjustment in amyloid‐positive MCI.^23^ Consistent with that, introducing age in sensitivity analyses reduced the t‐tau main effects and the PRS*t‐tau interaction on sTREM2. In contrast to PRS, which represents stable genetic predisposition independent of age, both immune activation biomarkers (e.g., sTREM2) and neurodegeneration biomarkers (e.g., t‐tau and NfL) typically increase with age.^58^ In this study, we found that CSF sTREM2 levels decrease with higher AD PRS_INFL_ in the context of elevated CSF t‐tau but show no significant association with NfL both when accounting for age and not.

Clusterin may be neuroprotective by limiting amyloid peptide misfolding and facilitating transportation across the blood–brain barrier,^59^ thus preventing Aβ aggregates.^60^ Positive associations between plasma clusterin and two AD PRSs, one general and one inflammation specific, have been observed,^61^ contrasting the negative association found here between AD PRS_INFL,_ clusterin, and t‐tau, as well as between sum PRS_IMMUNE_, clusterin, and NfL. In our study we focused on preclinical and prodromal AD groups. Morgan et al. carried out a smaller study, and involved participants with established AD dementia.^61^ Another important factor that may explain the different associations between the inflammatory genotype and clusterin levels is the type of biological fluid analysed. We measured clusterin in CSF rather than plasma. A meta‐analysis observed that plasma clusterin concentrations were increased in people with AD dementia, but not in people with MCI or other dementias.^62^ Thus, the lower sensitivity of plasma clusterin in the predementia stages would putatively not yield between‐group differences in our early preclinical and prodromal cases. Additionally, clusterin is produced in peripheral organs like kidney, liver, heart and testis^63^ and factors such as sex, obesity, systemic inflammation and atherogenic components of the lipid profile in AD patient can influence plasma clusterin concentrations,^64^, ^65^ thereby potentially impacting the results.

Fractalkine, also known as CX3CL1, is important for neuron‐microglia communication, working on its receptor predominantly found on microglia. Fractalkine exerts neuroprotective effects by reducing the expression of pro‐inflammatory genes in activated microglia and decreases tau pathology.^66^, ^67^, ^68^ Although sTREM2 and fractalkine did not present significant associations with t‐tau in the sum PRS_IMMUNE_ analyses, it is interesting to note the contrasting trends observed. Higher AD PRS_INFL_ was associated with lower concentrations of sTREM2 and fractalkine. Based on the protective roles of sTREM2 and fractalkine, this might be suggestive of unfavorable genetic predispositions in the form of high AD PRS_INFL_. In contrast, higher sum PRS_IMMUNE_ was associated with higher concentrations of sTREM2 and fractalkine, indicating a potential protective immune response. This suggests that higher sum PRS_IMMUNE_ may represent the beneficial effects of enhanced immune activation, whereas higher AD PRS_INFL_ may represent the detrimental ones, warranting further investigations to disentangle the diverse effects of genetic factors on innate immune response in predementia AD cases.

Under neuroinflammatory conditions YKL‐40 is primarily produced by astrocytes, with a smaller contribution from microglia.^69^ YKL‐40 has been observed around amyloid plaque^70^ and increasing concentrations are predictive of disease progression.^71^ Our findings are aligned with this: with more neurodegeneration (NfL) a positive association is observed between AD PRS and YKL‐40. A genetic polymorphism that results in lower YKL‐40 concentrations is associated with slower AD progression in humans.^72^ When examining the two immune activation PRSs, we found no significant associations with YKL‐40, with or without neurodegeneration.

Note that APOE was excluded from all the PRSs since a PRS in the context of AD will only be relevant if it adds extra information beyond the known and strong influence of APOE alone.^73^

The sample size used in this study presents both strengths and limitations. We restricted analyses to the full cohort (n = 294) to maintain power, as 29.9% were Aβ positive and 52.7% cognitively unimpaired (RaC and SCD), making stratified analyses underpowered. As discussed above, when analyzing associations in the full cohort, including age as a covariate may inadvertently adjust for disease stage, because older participants are more often cognitively impaired and Aβ positive. We mitigate this by prespecifying age‐excluded primary models and reporting age‐adjusted sensitivity analyses; nevertheless, residual confounding by age/stage cannot be fully excluded. A key strength of this study is the quality of information within our data (polygenic risk scores, neurodegeneration markers, cognitive status, and CSF innate immune activation markers) which allows us to explore complex associations within a relatively large sample size. Nevertheless, future research with larger cohorts and diverse populations, enabling adequately powered stratified analyses, will be essential to validate and further our understanding of the genetic underpinnings of immune responses in AD.

## 5 CONCLUSION

This study hints to a link between genetic predisposition for immune and specific immune activation markers in CSF during early stages of neurodegeneration. Our findings suggest that specialized PRSs like AD PRS_INFL_ and sum PRS_IMMUNE_ capture relevant and different genotype–phenotype associations related to immune activation in AD, that are not captured by standard AD PRSs. This research highlights the necessity of considering specific genetic predispositions when evaluating immune activation phenotypes in preclinical and prodromal stages of AD.

## AUTHOR CONTRIBUTIONS

Lene Pålhaugen and Kaja Nordengen developed the PRSs together with Francesco Bettella and Shahram Bahrami. Kaja Nordengen, Lene Pålhaugen, Bjørn‐Eivind Kirsebom and Stephanie Knudtzon conceived the present idea and designed the analyses. Stephanie Knudtzon performed the analysis and designed the figures with significant help from Bjørn‐Eivind Kirsebom. Stephanie Knudtzon wrote the main draft of the manuscript and interpreted the results in consultation with Kaja Nordengen and Bjørn‐Eivind Kirsebom. All authors have contributed with data collection, provided critical feedback, and helped shape the manuscript. Berglind Gísladóttir has analyzed all CSF biomarkers. KN supervised the project. Tormod Fladby is the leader of the DDI project. All authors have read and approved the final manuscript.

## CONFLICT OF INTEREST STATEMENT

B.E.K. has served as a consultant for Biogen and on an advisory board for Eisai and Eli Lilly. T.F. has served as a consultant and at the advisory boards for Biogen, Novo Nordisk, Eli Lilly, Roche and Eisai. P.S. has served as a consultant for Roche. R.E.S. has served on an advisory board for Eisai and as local PI on GSK 219867. O.A.A. is a consultant to Cortext.ai and Precision Health AS, and has received speaker's honorarium from Lundbeck, Janssen, Eisai and Lilly. All other authors declare that they have no competing interests. Author disclosures are available in the Supporting Information.

## ETHICAL APPROVAL

The study is approved by the regional medical research ethics committee. Written consent was given by all participants before taking part in the study. The study was conducted in line with the guidelines provided by the Declaration of Helsinki of 1964, 2013 revision, and the Norwegian Health and Research Act.

## Supporting information



Supporting Information

Supporting Information

Supporting Information

## Data Availability

Data from the DDI cohort are secured at Services for sensitive data (TSD) at the University of Oslo (UiO) and not publicly accessible. Nonetheless, pseudonymized data from this research may be provided by the corresponding author upon justified request.
